# Autism Spectrum Disorder and Fetal Alcohol Spectrum Disorder: A Literature Review

**DOI:** 10.3390/brainsci12060792

**Published:** 2022-06-16

**Authors:** Barbara Carpita, Lavinia Migli, Ilaria Chiarantini, Simone Battaglini, Clara Montalbano, Claudia Carmassi, Ivan Mirko Cremone, Liliana Dell’Osso

**Affiliations:** Department of Clinical and Experimental Medicine, University of Pisa, 56126 Pisa, Italy; lavinia.migli@gmail.com (L.M.); chiarantini9@gmail.com (I.C.); battaglini.simone@gmail.com (S.B.); montalbanocl@gmail.com (C.M.); ccarmassi@gmail.com (C.C.); ivan.cremone@gmail.com (I.M.C.); liliana.dellosso@med.unipi.it (L.D.)

**Keywords:** autism, autistic traits, fetal alcohol spectrum disorder, fetal alcohol syndrome, neurodevelopmental disorder

## Abstract

Fetal alcohol spectrum disorders (FASD) are a group of conditions associated with the effects of prenatal alcohol exposure and characterized by somatic and neuropsychological alterations. On the other hand, autism spectrum disorder (ASD) is characterized by a multifaceted neurobehavioral syndrome. Since alcohol can affect every stage of brain development, some authors hypothesized that in utero alcohol exposure might be linked to an increased risk of ASD in subjects with genetic vulnerability. The present review aimed to summarize the available literature on the possible association between FASD and ASD, also focusing on the reported clinical overlaps and on the possible shared pathogenic mechanisms. Studies in this field have stressed similarities and differences between the two conditions, leading to controversial results. The available literature also highlighted that both the disorders are often misdiagnosed or underdiagnosed, stressing the need to broaden the perspective, paying specific attention to milder presentations and sub-syndromic traits.

## 1. Introduction

Fetal alcohol spectrum disorders (FASD) are a group of conditions associated with the effects of prenatal alcohol exposure and characterized by several kinds of impairments: distinctive facial characteristics (palpebral fissures, a smooth philtrum, a thin upper vermillion border, maxillary hypoplasia), cardiac defects, and growth retardation. Knowledge about FASD is still in its infancy. FASD is a condition of recent definition and, in the Diagnostic and Statistical Manual of Mental Disorders (DSM-5), is still included among the conditions for which more research is needed [1]. In addition, the broad FASD category features conditions with different grades of severity. When fetal alcohol syndrome (FAS), the most severe form of FASD, was firstly identified, the newborns diagnosed with FAS shared some distinctive physical characteristics [2]. However, further studies progressively highlighted that alcohol-induced alterations may be expressed by a broader range of symptoms and traits than previously thought. In particular, FAS manifestations may vary depending on the dosages, the trimester of exposure, the mother’s food intake and genetic factors. For example, not all the children with prenatal alcohol exposure show typical facial characteristics [2]. Noticeably, the most critical effects of in utero alcohol exposure were reported to be those on the fetal brain; alcohol may affect every single stage of brain development, from neurogenesis to myelination [2]. Initially, confirmed maternal alcohol consumption was not needed for the diagnosis of FAS. On the other hand, the term Partial FAS was used when the newborn had only some characteristics typical of the syndrome, but there was a confirmed anamnesis of maternal alcohol consumption. It should be noted that the term FASD could be considered an umbrella definition that includes different kinds of alcohol-related conditions with different grades of severity [2]. However, the range of conditions related to in utero alcohol exposure may be even broader, eventually including more hidden outcomes, such as sudden infant death [3]. Prevalence ranges for FASD greatly vary depending on the studies. When all the possible manifestations of prenatal alcohol exposure are taken into account, the prevalence is consistently higher. In one study, the estimated prevalence of FASD was approximately 1 per 100 live births [4]. The prevalence reported is different depending on the specific population studied and on the socioeconomic status of the subjects. In a retrospective case-control study, 80.3% of 132 identified FAS cases were Native Americans, while 18.2% were Caucasian [5]. Native Americans showed a greater tendency towards binge drinking, while Caucasians reported different drinking behaviors: peak blood alcohol levels seem to be a critical factor for the effect of alcohol on neurodevelopment [5,6]. Another point of heterogeneity in prevalence studies is the method of assessment. Intriguingly, May and Gossage [7], using an active case assessment method through in-school screening and diagnosis, reported a higher FASD prevalence with respect to other studies (2–5%), confirming that alcohol could be considered the most frequent preventable cause of mental retardation [8]. According to a systematic review, the general population prevalence of FAS is 1.5 cases per 1000 newborns, but this data represents only the tip of an iceberg since the prevalence ratio of FASD to FAS is generally believed to be around 10 to 1 [9]. Unfortunately, drinking during pregnancy, and even binge drinking, is still quite common, and the need for universal screening with respect to alcohol exposure was previously stressed in the literature [9,10].

Fatty acid ethyl esters (FAEEs) have been studied as a marker of in utero alcohol exposure: when the fetus is exposed to alcohol, FAEEs can be found in the meconium. A review on this topic reported a 22.6% prevalence of high levels of FAEEs in the meconium [3]. Hutson et al., setting a cut-off of 2 nmol/g for high-risk alcohol exposure, reported that 44% of the meconium samples were above this threshold [11]. A study performed in Canada found that 2.5% of the meconium samples showed high levels of alcohol exposure: interestingly, this rate was 5 times higher than the rate of high-risk alcohol exposure detected by means of questionnaires [12]. In a Texan study, an ethanol metabolite, phosphatidylethanol, was measured in 1000 newborns dried blood spot samples in order to evaluate the prevalence of prenatal alcohol exposure, reporting a rate of 8.4% [13]. However, more research on this topic is needed: while using FAEE as a biomarker could be useful for identifying alcohol-exposed newborns, this method can only be used after childbirth and cannot provide information during the first trimester. The first trimester is very important because in this timespan, teratogen exposure may critically affect the child, and, on the other hand, women may drink more alcohol, being not aware of their pregnancy [2,3].

Besides environmental factors, such as the levels of alcohol exposure or the drinking pattern of the mother, another important risk factor for FASD is the amount of genetic susceptibility towards the teratogenic effect of alcohol. A study conducted on twins showed a 100% rate of concordance for FAS in monozygotic subjects, but only a 63% concordance rate for dizygotic ones [14].

Autism spectrum disorder (ASD) is a multifactorial condition characterized by deficits in social skills, narrow interests and repetitive behaviors. The definition of ASD includes conditions with different levels of severity, with or without intellectual impairment or language development alterations. Genetic factors play a crucial role in ASD, and siblings of ASD individuals were reported to show a 50 times increased risk of developing ASD with respect to the general population [15,16,17]. However, current evidence also supports the role of environmental factors, in particular during intrauterine life, such as maternal food intake, maternal inflammation or air pollution [18,19,20,21]. In particular, different environmental factors may interact with multifaceted genetic underpinnings (featuring multiple susceptibility genes) in shaping the variety of ASD manifestations. The specific grade of severity, the location and timing of the neurodevelopmental alteration may lead to different psychopathological trajectories [22,23,24].

To date, studies about the possible association between FASD and ASD are still limited in number, leading to controversial results [18]. A systematic review and meta-analysis reported that ASD seems to be present in 2.6% of FASD children, which is a rate almost two times higher than that reported in the general US population [25].

Prenatal alcohol exposure can result in impaired brain function, affecting every single stage of neurodevelopment [2,5]. Therefore, it is possible that high levels of in utero alcohol exposure may be linked not only to FASD, but to an increased risk of ASD as well.

In this framework, the aim of this work was to review the available studies on the relationship between ASD and in utero alcohol exposure, also focusing on the reported overlaps between ASD and FASD and on the possible shared pathogenic mechanisms. In particular, we aimed to summarize the findings from previous studies, also focusing on the possible relationships between different works, in order to evaluate if some conclusion in this field may be reached on the basis of the available literature or to identify the need for additional research.

## 2. Methods

A comprehensive literature search was performed in order to identify all studies focused on the topic of the relation between ASD and FASD. The literature search was led on multiple databases (Pubmed, Scopus, Web of Science) using the following keywords: alcohol embryopath*, alcohol* related* birth defect*, arbd, arnd, fae, fas, fasd, fetal alcohol syndrome, fetal alcohol spectrum disorder, autism spectrum disorder*, autism, neuro-developmental disorder, biological correlates, pathogenic mechanisms. We included studies published from 1980 to 2022 in the English language and in peer-reviewed journals. Studies were excluded if not written in the English language; case reports were also excluded. Among the identified works, those actually focused on the investigated topic have been included in the review.

## 3. Prevalence Studies on the Link between ASD and Maternal Alcohol Consumption or FASD

### 3.1. Studies Focusing on In Utero Alcohol Exposure among Subjects Already Diagnosed with ASD

A case-control study compared data collected from 102 autistic individuals and 106 developmentally disabled controls of similar age, sex ratio and non-verbal intelligence, failing to find a significant association between maternal alcohol consumption during pregnancy and ASD. However, mothers of the controls reported a significantly higher alcohol intake during pregnancy [26]. Another case-control study examined prenatal and perinatal risks among three different subgroups: subjects with ASD (121 individuals), subjects with pervasive developmental disabilities (75 individuals) and controls (311 individuals). A significant association between alcohol exposure and ASD was not found. However, the pervasive developmental disorder group was associated with a higher smoking exposure during pregnancy [27].

The SEED study (Study to Explore Early Development) is a multi-centric case-control study comparing alcohol exposure in different time periods in a group of subjects with ASD (684 children), a group of individuals with non-ASD developmental disorders (869 children) and 962 controls. The presence, timing and levels of mothers’ alcohol consumption were collected through self-report assessment methods. The study highlighted no association between low levels of maternal alcohol use and ASD or non-ASD developmental disorder. An inverse association was instead found in the preconception period and in the third trimester. This inverse association may be linked to the retrospective design of the study: participants were asked about their alcohol consumption during pregnancy two to five years after the event. In addition, the self-reported assessment may be biased by the possible tendency toward under-reporting alcohol use during pregnancy, especially in the case of mothers of unhealthy children. Other possible explanations may be linked to genetic causes or to the fact that healthy women may be more prone to occasionally drink alcohol than women with complicated pregnancies or with unhealthy children from previous pregnancies [28]. Studies are summarized in Table 1.

### 3.2. Studies on the Prevalence of ASD Diagnosis in Children with Different Levels of Alcohol Prenatal Exposure or FASD

A prevalence study realized in Saskatchewan, a Canadian region with a high prevalence of FAS, detected an ASD prevalence of 3.4% (n = 7) in a clinical sample of 207 ascertained cases of FAS [29].

Aronson et al. performed a follow-up evaluation of 24 children (age range 11–14 years) whose mothers abused alcohol during pregnancy. Among them, 2 subjects were diagnosed with Asperger syndrome and one with an autistic-like condition. According to the authors, the frequency and severity of the clinical correlates were associated with the level of in-utero alcohol exposure [30]. In addition, the study reported that early fostering did not eliminate the effect of in utero alcohol exposure, stressing that neurodevelopmental and behavioral outcomes of alcohol abuse during pregnancy were related to biological and not to psychosocial factors. Moreover, the authors pointed out that children whose mothers discontinued alcohol consumption in the second and third trimesters did not show developmental alterations, suggesting that these months could be crucial for the development of the executive functions that result to be impaired in ASD [30].

O’Connor and colleagues selected 23 children with in utero heavy alcohol exposure. Among them, 17 subjects were affected by alcohol-related neurobehavioral disorders, while the others reported a diagnosis of FAS/partial FAS. The prevalence of psychiatric disease in this group was evaluated, and the most frequent diagnosis was mood disorder, but there was 1 child (4% of the sample) with pervasive developmental disorder [31]. Green et al. investigated 97 FASD subjects and 92 controls with the aim of evaluating the prevalence of executive dysfunctions in FASD children. Subjects were assessed using neuropsychological tests of the Cambridge Neuropsychological Test Automated Battery. The study also considered the presence of comorbidities. A diagnosis of ASD was detected in 2 FASD subjects (2.3% of the clinical sample), while no ASD cases were reported in the control group [32]. Bell et al. performed a retrospective review on charts, examining 1063 cases referred to two FASD clinics [33]. They only selected cases with a confirmed diagnosis, resulting in a final sample of 425 patients. While the main goal of the study was to evaluate if FASD children reported a higher prevalence of epilepsy/seizures, the authors also highlighted that 8 children (1.9%) had a confirmed autism diagnosis.

Landgren et al., in a study on 71 children adopted from Eastern Europe, revealed a 52% rate of subjects (n = 37) with FASD. Authors reported that at least 34% of the biological mothers abused alcohol during pregnancy, but this data was probably underestimated. Autism was diagnosed in 9% of the sample, according to DSM-IV criteria. While the authors did not recruit a control group, the prevalence rates of ASD were strikingly higher than those reported in the general population [34]. Eliasen et al. led a study in a large cohort of 80,552 Danish children and mothers who were followed during pregnancy and until the child was around 7 years old. According to the Danish Central Psychiatry Register, 401 children were diagnosed with ASD and 157 with infantile autism. The study did not report a significant association between ASD and average alcohol consumption, while binge drinking during pregnancy was associated with a lower risk of ASD. Despite the large sample size and the prospective design of the study, it should be taken into account that the assessment of alcohol during pregnancy through self-report methods may have affected the results [35]. In addition to the issue of self-report assessment, the same authors stressed that other confounding factors might be responsible for the reported lower risk of ASD among mothers with binge drinking habits, such as the lower consumption of alcohol among women at high risk for reproductive failure [35]. Stevens and colleagues compared 25 children with FASD and 17 controls. They found that FASD children showed significantly higher autistic traits, such as poorer social skills, more behavioral problems and higher scores on autism-related psychometric instruments [36]. Chasnoff et al. evaluated the rate of missed diagnoses/misdiagnosis of FASD in 547 children. Among 156 subjects correctly diagnosed with FASD, 5% of the sample reported a comorbid autism/pervasive development disorder. This study revealed a high rate of FASD missed diagnosis (80.1%) and of change in the diagnosed mental condition, thus stressing the importance of an extensive multidisciplinary evaluation [37].

A study performed in a small sample of 21 subjects with FASD suggested instead an association between heavy alcohol exposure and ASD, reporting that 72% of FASD individuals (n = 16) met the International Classification of Diseases (ICD)-10 criteria for ASD. The authors also compared the group of ASD subjects with a control group of ASD children without alcohol exposure, reporting differences in ASD manifestations between prenatally alcohol-exposed and non-exposed ASD groups. In particular, alcohol-exposed ASD subjects seemed to show a passive or bizarre type of social interaction (with a higher tendency to be bullied and to report a lack of common sense) rather than aloofness and to show milder coordination deficits with respect to non-exposed ASD subjects [38]. However, due to the small sample size, further research is needed to confirm the findings of this study. Another work from Mukherjee and colleagues investigated the possible impact of neglect and prenatal alcohol exposure on child neurodevelopment. The clinical sample was composed of 99 subjects diagnosed with FASD, aged from 6 to 26 years. A 68% rate of ASD and/or social communication disorder was reported. No significant difference was found between subjects exposed or not to neglect [39].

Gallagher et al. reported data from “The Millennium Cohort Study”, a retrospective analysis performed on a cohort of 18168 mother–child pairs. Information about mothers’ alcohol consumption and about the presence of ASD in the offspring was collected via parental questionnaires: mothers’ alcohol intake during pregnancy was classified as light, moderate or heavy. The study failed to find a significant association between ASD diagnosis among children and alcohol intake. Only a non-statistically significant association was found in the case of heavy alcohol intake. However, as in the case of previous investigations featuring self-report assessment methods, since the information retrieval was made retrospectively, a certain amount of recalling biases and under-reporting for social desirability should be considered when evaluating results from this study [40]. Studies are summarized in Table 2.

## 4. Similarities between FASD and ASD Children

Besides epidemiological studies, other research focused instead on evaluating the similarities in the presentation of FASD and ASD, highlighting a set of psychopathological overlaps. FASD children were often reported to show poor social abilities and inaccurate judgment in social situations [41]. However, other authors stressed that FASD individuals seem to desire social interactions, being characterized by a lower impairment of empathy and social skills as well as by a higher ability to use non-verbal language, start a conversation or participate in social activities [2,42]. On the other hand, showing inappropriate social behaviors is not equivalent to meeting an ASD diagnosis, and social difficulties are also reported in several other mental disorders. In conditions such as attention deficit hyperactivity disorder (ADHD) or social anxiety disorder, the social impairment may arise as a consequence of the hyperactivity/inadequate social behavior or of the fear of judgment, respectively, while in ASD, the impairment of social communication and interactions is one of the core features of the clinical condition [42]. In this framework, further studies should evaluate if the presence of altered social skills may be considered a core symptom of FASD or an associate condition. Noticeably, children with FASD were reported to show poor executive functions and an altered theory of mind, which is one of the features at the basis of ASD-like relational impairment [43,44,45]. Theory of mind and executive functions are believed to be strongly related, while poor executive functions might underlie theory of mind deficits [45].

Intriguingly, the worse social skills reported in FASD individuals seem to be present independently from the intelligence quotient (IQ), as reported by studies that compared FASD children and IQ-matched controls [36,46,47], suggesting that the social deficits in this population may not be exclusively related to intellectual impairment. In particular, Thomas and al. reported that FASD children showed worse social skills when controls were matched for verbal IQ, a feature specifically associated with social abilities [46]. While some authors pointed out the potential role of a dysfunctional environment in shaping poor social abilities in FASD children, it should be noted that in this population, early fostering seemed to not eliminate the psychopathological consequences of in utero alcohol exposure [30].

In addition to social impairments, individuals with FASD were reported to show other ASD-like features, such as poorer adjustment abilities, altered response to sensory inputs, and repetitive interests/behaviors [36,48,49].

Stevens et al. led a study comparing FAS subjects and controls, asking parents to rate their children by means of the Social Skills Improvement System, an instrument for evaluating social skills, specific behavioral patterns and ASD-like features. The authors highlighted that FASD children showed more ASD-like traits than controls, such as poor social interaction skills. In particular, they showed difficulties in understanding turns during conversations or in changing routine, with a higher tendency towards an insistence on sameness and social withdrawal [36]. Some evidence in this field also comes from animal models: alcohol exposure during synaptogenesis was reported to trigger massive apoptotic neurodegeneration in rat brains, especially in cerebellar neurons, a finding which might be in line with the decreased number of Purkinje cells found in ASD brains [21,50]. Middleton and al. [51] studied ethanol teratogenic effects in a rat model, reporting social avoidance among rats exposed to alcohol. These authors also hypothesized that the effects of alcohol on social behaviors might be shaped by the specific timing of the exposure and by the consequent changes in different brain areas, including the ventral striatum and the amygdala [51].

Finally, ASD and FASD seem to also share a set of comorbid symptoms and conditions. ASD children were often reported to show more externalizing behaviors [52,53], hyperactivity and attention deficits, in particular when not focused on their topic of choice [53], together with a high prevalence of comorbid ADHD symptoms and traits [54]. Similarly, FASD children often showed externalizing behaviors and a comorbid ADHD diagnosis [25]. In addition, ASD, ADHD and FASD seem to share an association with increased deficits in manual dexterity. However, a study that compared manual dexterity in these conditions revealed in ASD children worse non-dominant hand dexterity and a significantly higher hand performance asymmetry with respect to FASD children [55].

Similarities between FASD and ASD subjects with respect to sleep patterns were also highlighted, such as shorter total night sleep duration and more night awakenings, with a global reduction of sleep efficiency. However, the structural neural damage underlying these sleep disturbances was hypothesized to be different in the two conditions [56]. Studies are summarized in Table 3.

## 5. Possible Shared Biological Underpinnings between FASD and ASD

### 5.1. Folate Levels

The crucial role of folate in neurodevelopment is well-known in the literature, and folate supplements are commonly used in order to prevent spina bifida in the fetus [57]. On the other hand, alcoholism is reported to be a common cause of folate deficiency [58].

While ethanol-related oxidative stress was considered to be involved in FASD pathogenesis, folic acid was instead implicated in reducing the severity of this damage [59].

A recent work analyzed the mechanisms through which formic acid and folic acid may play a role in the pathogenesis of FASD. Higher levels of formic acid are typically detectable among alcoholic women. Formic acid is able to cross the placenta barrier, thus exerting its neurotoxic potential, also linked to the induction of neural death, on the fetus [60]. On the other hand, folate levels are usually lower among alcoholic patients [58]. During pregnancy, folate exerts a crucial role, being needed to increase DNA production and cell division. Folate levels are reported to be generally twice as high in cord blood as maternal blood, but this ratio was revealed to be reversed in alcoholic mothers, possibly because folate transporters in the placenta seem to be down-regulated in these patients [61]. The combination of higher formic acid and lower folic acid is of particular significance because the neurotoxic effect of formic acid may be counterbalanced by folic acid, enhancing formic acid elimination [60]. Moreover, low folate levels and high formic acid levels may play a role in alcohol-related brain damage through oxidative stress and mitochondrial dysfunction [60].

At the same time, several findings stressed a possible association between altered folate levels and ASD. Previous studies pointed out a reduction of ASD risk among women using folate supplements [18,62,63], as well as altered folate and homocysteine levels in ASD children and an improvement of ASD symptoms with folate supplementations [64]. However, not all the studies confirmed these results, and a conclusive agreement on this matter was not reached [65,66]. Further authors highlighted an association between increased ASD risk and the presence in mothers and/or in children of genetic variants linked to less efficient folate metabolism. In particular, the role of methylene tetrahydrofolate reductase (MTHFR) polymorphisms in ASD risk was stressed in the literature [62,64]. Other factors linked to folate deficiency may be immunological alterations, e.g., the presence of folate receptor alpha (FRα)-autoantibodies found in ASD children and their relatives may be responsible for folate deficiency in cerebrospinal fluid due to the ability of autoantibodies to prevent folate transportation across the blood–brain barrier [64,67].

On the other hand, a recent Swedish study examined serum nutrient levels at week 14 in 100 women with ASD children and in 100 matched controls. A total of 62 metabolic bio-marker were measured, including folate, vitamin B and vitamin D [66]. Results highlighted a positive, although weak, association between high maternal folate levels and increased rates of ASD, while inflammation markers did not show an association with ASD occurrence.

The high heterogeneity reported in the results on this topic may depend on several epidemiological factors and on sample selection biases, such as folate level variability in the chosen populations, the uselessness of folate supplementation above a certain threshold or the different prevalence of less efficient genes related to folate metabolism in different samples [66,68].

Folate may be involved in ASD pathophysiology in different ways, being able to affect immune system modulation, homocysteine metabolism, oxidative stress balance and gene expression through DNA methylation. However, altered folate levels in ASD individuals may also be considered a consequence of the neurodevelopmental disorder or as a concomitant condition, eventually with shared pathophysiological mechanisms [66,68].

### 5.2. Epigenetics

During prenatal and early postnatal periods, brain development is vulnerable to injuries by environmental factors, including alcohol. In this framework, alcohol was hypothesized to induce epigenetic changes, which might be associated with detrimental effects on the fetal brain [69]. On the other hand, some disorders such as ASD, Rett syndrome and Fragile X syndrome were reported to be associated with epigenetic deregulation [69,70].

Several studies showed that the expression of genes related to development can be modified by epigenetic mechanisms; one of the altered epigenetic mechanisms involved in ASD is impaired DNA methylation [69]. Noticeably, gestational alcohol exposure was shown to induce aberrant changes in the methylation profile of over 1000 genes in mouse embryos at the early neurulation stage. These alterations were associated with disruptions in neural specification and growth [71]. Alcohol exposure was associated with hypo-methylation of fetal DNA, possibly due to a reduced level of DNA methyl transferase [72,73]. Another study highlighted aberrant methylation in adult mice prenatally exposed to alcohol; over 30 genes were found to have an altered expression [74]. In addition, Hicks et al. revealed that ethanol enhanced DNA methylation of genes related to cell cycles, affecting cell cycle regulation and nervous system growth [75].

A link was reported between alcohol exposure during development and alterations in gene expression of DNMT1 (coding for DNA methyl transferases 1), DNMT3a and MeCP2 (coding for methyl CpG binding protein 2) [76]. This data is of particular interest because the hypo-methylation associated with altered DNMT1 expression was reported to be involved in the pathophysiology of long-term potentiation and synaptic function impairment, eventually leading to alterations in neuronal cells [76]. Moreover, altered levels of DNMT, associated with alcohol exposure during the third trimester of pregnancy, seemed to imply, in turn, changes in MeCP2 expression [77,78,79].

On the other hand, a correlation was found between ASD and mutations of MeCP2, whose protein product plays a key role in regulating other genes linked to neurodevelopment [77]. Some individuals with childhood autism show MeCP2 mutations, while in tissue samples of ASD brains, a reduced MeCP2 protein expression was frequently highlighted, linked to increased methylation of MeCP2 promoter [80].

Alcohol exposure appears to inhibit the differentiation of neural stem cells and may reduce the expression of genes related to neurodevelopment (Ngn1, Ngn2, Sox 5, and Sox 7), neural growth factors (Igfbp2 and Efemp1) and cell cycle regulation (Clk1, CLk4, and Ndrg1) [81,82].

In conclusion, epigenetic studies seem to support the hypothesis that alcohol exposure may enhance some of the epigenetic alterations which were reported to be associated with neurodevelopmental disorders such as ASD. However, studies in this field are still limited: a better understanding of epigenetic mechanisms could shed light on the pathophysiology of both ASD and FASD.

### 5.3. Inflammation

Recently, several studies highlighted a possible involvement of neuroinflammation and altered immune response in ASD pathophysiology [20,21,22,83]. While ASD patients often report autoimmune disorders in comorbidity, lower levels of anti-inflammatory cytokines and increased levels of pro-inflammatory cytokines were repeatedly highlighted in this population [21]. Altered levels of specific cytokines have been related to the severity of ASD symptoms [21,83]. Activation of microglial cells and reactive gliosis were also highlighted in ASD brain specimens [21,84], as well as increased levels of the beta-amyloid precursor protein, a potential marker of axonal injury and neuroinflammation [21]. Some authors also hypothesized a link between ASD pathophysiology and an imbalance between the neuroprotective and neurotoxic branch of the kynurenine pathway of tryptophan metabolism, featuring altered levels of kynurenic and quinolinic acid. An alteration of this metabolic flux may lead to increased glutamatergic transmission and excitotoxicity in ASD subjects, with subsequent impairments in neural functions as well as increased activation of the immune system and inflammatory processes, which, in turn, may contribute to the imbalance of the kynurenine pathway [21,85]. In addition, ASD was linked to increased oxidative stress by several studies, with reduced levels of anti-oxidant species, such as glutathione [21,85]. Conditions linked to increased inflammatory state and oxidative stress exposure in an intrauterine environment, such as maternal obesity and diabetes, were reported to be associated with a higher prevalence of ASD in the offspring [20]. However, it should be noted that despite the large number of studies in this field, the actual role of inflammation in ASD remains unclear, and further studies are needed to clarify whether the altered inflammatory state, immune response and oxidative stress balance in this population should be considered as a cause, a consequence or a parallel process with respect to the neurodevelopmental condition [21].

In the field of FASD, the role of alcohol exposure in the enhanced production of reactive oxygen species (ROS) during fetal development and in adult life was frequently reported: alcohol-related increases in ROS may also lead to an increase in apoptotic processes [86,87]. ROS are actually unstable substances involved in the damage of macromolecules. The production of hydrogen peroxide, superoxide anions and hydroxyl radicals cause alteration in signal transduction pathways, leading to cell death and teratogenesis [87,88,89]. In this framework, the teratogenic effects of ROS were demonstrated in brain cells. Free radicals may cause an imbalance in cell redox state and, through lipid peroxidation and DNA damage, lead to apoptosis [88,89] or autophagy [90]. Moreover, alcohol intake seems to also damage cells indirectly through acetaldehyde, the proximate metabolite of ethanol. The production of acetaldehyde implies an increase in the activity of the respiratory chain and, therefore, in the ROS production [88,91]. In line with this data, high levels of acetaldehyde in mouse midbrain cells were reported to be linked to cytotoxicity and apoptotic neurodegeneration [92].

Alcohol intake during pregnancy is also supposed to affect the fetal immune system, causing an increase in pro-inflammatory cytokines and a reduction in lymphocyte proliferation and maturation [92,93]. Ethanol was reported to promote increased IL-1β, IL-1α, TNF-α and CCL2 in the brain [93,94,95,96], eventually leading to neuroinflammation and neurodegeneration [95]. Reduced gestational production of alpha-fetoprotein was found to be associated with alcohol consumption and increased levels of IL-6 during pregnancy. Intriguingly, IL-6 is one of the cytokines typically found to be increased among ASD children, and a possible correlation was reported between reduced alpha-fetoprotein levels in pregnant women and the presence of ASD in the offspring [21,97,98]. Finally, alcohol intake during pregnancy was reported to be associated with alterations in neurotransmission, specifically affecting L-glutamate release in the fetus’s hippocampus [98] and eventually delaying neuronal growth [98,99].

### 5.4. Dysbiosis

The effect of alcohol in promoting dysbiosis and gut permeability is well-known in the literature. Alcohol may alter the immune activity and the mucosal tight junctions in the gastrointestinal tract, leading to an alteration of the gut barrier, which allows microbial-dependent products (MDPs), such as fatty acids, indoles, bile acids and phytochemicals, to pass into the circulation [100,101,102]. Subsequently, MDP can also cross the placenta, entering fetal circulation, where they could directly impact fetal development.

Alcohol was also reported to alter the composition of gut microbiota, promoting the growth of Gram-negative facultative anaerobes that produce exotoxins such as lipopolysaccharide [102,103,104]. These products, through toll-like receptors (TLR), were hypothesized to enhance inflammatory activity, fibrosis and cell death, possibly being one of the mediators of alcohol-related organ damage. In mouse models of prenatal alcohol exposure, a loss of function in TLR4 also seemed to reduce the neuro-inflammatory response, as well as to protect from the development of social behavior deficits and cognitive impairment [104,105,106,107].

However, to date, there is no evidence that dysbiosis and microbiota dysfunction may contribute to the development of FASD, and the specific mechanisms through which these alterations may be involved in FASD pathophysiology need to be further investigated [107].

Noticeably, microbiota and the central nervous system (CNS) show reciprocal interactions, being able to influence each other through several metabolic mechanisms (including tryptophan metabolism). Moreover, microbiota and CNS may also communicate through enteric nervous system or hypothalamic pituitary adrenal axis modifications and through the modulation of the immune system [21]. Considering these findings, the presence of a “gut-brain-axis” was frequently stressed in the literature, in which the immune system may play a key mediating role [21].

In this framework, besides the above-reported associations between ASD and altered immune response, a link between microbiota composition and ASD was hypothesized in the literature [21]. The interest in the role of microbiota in ASD originally arose from the observation of an increased prevalence of gastrointestinal problems in ASD children [21,108]. In rodent models, a link between autistic-like behaviors and microbiota alterations was highlighted [21,108]. Several studies focused on microbiota composition in this population, often reporting specific microbiota profiles when compared with controls. However, research in this field is highly heterogeneous with respect to sample selection and methods of investigation, sometimes leading to controversial results [21].

## 6. ASD and FASD: Two Underdiagnosed Conditions

When considering the possible overlaps between ASD and FASD, it should be noted that both the disorders were supposed to often be underdiagnosed, in particular when their presentation is of milder severity [1,24]. Recently, increasing attention was paid to milder ASD manifestations without language or intellectual impairment, as well as to the broader spectrum of subthreshold autistic traits [17,24]. Due to the lack of language or intellectual impairment during childhood/adolescence, milder forms of ASD may come to clinical attention only during adulthood, when the subjects develop other psychiatric disorders in comorbidity, for which ASD is considered a risk factor [24]. The presence of significant, although subthreshold, autistic traits was initially noticed among first-degree relatives of ASD children [17,24]. On the other hand, autistic traits were also reported to be distributed in a continuum in the general population and to be particularly frequent in some high-risk groups as well as among clinical groups of psychiatric patients affected by other psychiatric disorders [17,24]. The interest in autistic traits lies in the fact that they were reported to also exert a significant impact on quality of life when subthreshold and to represent a risk factor for developing other psychopathological conditions, as well as suicide thoughts and behaviors [17,24].

On the other hand, FASD diagnosis also implies several challenges and, as a consequence, is currently believed to be underperformed in general treatment settings [109]. In particular, while parents’ assessment of child development is usually considered reliable, this may not be the case for parents with high alcohol intake or other substance abuse [110]. A particularly critical issue is represented by the lack of generally accepted recommendations for FASD diagnosis, especially with respect to milder forms such as alcohol-related neurobehavioral disorders (ARND) [111].

When evaluating a possible FASD diagnosis, the clinicians should consider several issues, including evidence of in utero alcohol exposure, the importance of genetic and environmental factors and the differential diagnosis between FASD and other neurodevelopmental conditions or potential comorbid disorders [109]. A previous study reported an 80.1% rate of missed FASD diagnoses in 547 children referred to a mental health clinic (125 out of 156 children and adolescents who met FASD criteria had never received the diagnosis before). Different causes might underlie this issue, including the fact that distinctive physical characteristics (such as facial dysmorphia and growth impairment) were not always present and the frequent comorbidity with neuropsychiatric disorders [37]. Moreover, often information about in utero alcohol exposure is missing [37,111]: alcohol consumption is commonly under-reported, especially in pregnant women [38,112]. Even in research settings, when the study design is retrospective, recalling and selection biases are likely to occur. Noticeably, the cases of high-level alcohol exposure detected through biomarkers such as FAEEs generally outnumber those detected by means of questionnaires [12]. Despite the above-reported difficulties in detecting FASD, early identification of FASD cases is highly recommended since an early intervention could allow reaching a better long-term outcome of the condition, reducing the impact on quality of life in terms of social, psychopathological and somatic consequences [37,113]. FASD subjects, even those with milder symptoms, showed an increased risk of experiencing troubled academic careers, comorbid mental disorders and alcohol/drug abuse, which further worsened their physical and mental condition [10,109,111].

In addition, while more attention should be paid to correctly detecting FASD cases of different severity, it should be noted that not all the symptoms reported by FASD children may be related to in utero alcohol exposure. As stressed above, subjects with FASD often show diagnostic overlaps and comorbidities with other conditions, which should be carefully considered in order to project targeted therapeutic strategies. Finally, as FASD children may live in a problematic familiar context, environment-related issues should also be evaluated and addressed [114].

In summary, the full-threshold presentations of ASD and FASD that come to clinical attention may be only the tip of an iceberg, while the broader, under-recognized sub-threshold spectra of both the conditions should be further explored in order to clarify possible overlaps from a psychopathological, epidemiological and pathophysiological point of views.

## 7. Discussion

As reviewed above, studies investigating the possible association between ASD and FASD or maternal alcohol exposure are still scant in number and feature heterogeneous protocols, leading to controversial findings [18]. Some of the available studies were conducted in small or very selected samples, limiting the extensibility of the results. In addition, research in this field is affected by the same critical limitation of all the investigations related to FASD, which is the difficulty in properly assessing the actual alcohol consumption during pregnancy. Alcohol exposure detected via questionnaires is likely to be scarcely reliable and generally under-reported [115]. Besides recall biases, women may minimize or lie about their alcohol consumption during pregnancy due to social desirability or fear of shame, in particular when the children show some symptoms [41]. In addition, as previously described, FASD diagnosis may be challenging, especially at birth or even later when symptoms are milder [35]. On the other hand, parents with heavy alcohol consumption may be less reliable in detecting and reporting anomalies in children’s behavior or may avoid medical consultations [35,36].

Another limitation of the available literature is the limited number of studies that properly differentiated subjects on the basis of levels of alcohol exposure before evaluating the association with ASD [36,39,41]. In addition, the difference between binge drinking and continuous alcohol consumption also needs to be specifically addressed with respect to the risk of FASD development [5,6,35,38]. Another important limitation is the extreme difficulty of leading studies focused on comparing the consequence of alcohol exposure in different trimesters of pregnancy. While this point may imply differences in the development of FASD itself, the issue of the ‘critical time window’ should be considered of utmost relevance in order to clarify the eventual presence of a link between ASD and alcohol exposure. Altered synaptogenesis seems to play a key role in the development of ASD, and the most relevant time for this process is the second trimester [116,117]. On the other hand, neurogenesis disruption also appears to be crucial in ASD pathogenesis [68,117]. Other evidence suggested that ASD features would be detectable since the first trimester: valproic acid, which has been linked to the development of ASD-like symptoms in children exposed to the drug during pregnancy, is supposed to exert its action around the time of neural tube closure [22,23,118]. Aronson et al. reported that mothers who do not drink alcohol in the second and third trimesters gave birth to normally developed children, stressing that these periods are crucial for the development of the executive functions typically impaired in ASD [30]. The SEED study [29] failed to find an association between low levels of maternal alcohol consumption and ASD. However, information about alcohol consumption was retrieved 2–5 years after delivery, exposing the study to recall biases. An inverse association was reported between ASD and alcohol consumption during the third trimester, which is difficult to explain. The authors hypothesized that healthy women might be more likely to occasionally drink alcohol than women with complicated pregnancies or with unhealthy children from previous pregnancies. In addition, several other confounding variables should be taken into account when evaluating the relationship between in utero alcohol exposure, FASD and ASD, including maternal age, ethnicity, smoking and dietary habits, as well as psychiatric and somatic comorbidities [38]. Noticeably, ASD seems to share genetic vulnerability with other psychiatric conditions such as anxiety and mood disorders, and also with alcohol use disorder (AUD) [119,120,121,122]. AUD and ASD were hypothesized to share some pathophysiological mechanisms, such as deregulation of serotonin and dopamine systems. In addition, AUD was reported to be more frequent in families with ASD children [23,120,122,123].

From a pathophysiological point of view, although, as reviewed above, an association between the two conditions on the basis of shared biological underpinnings is conceivable, there is still a lack of studies in this field. On the other hand, according to the available literature, the shared biological alterations might also be considered more broadly related to the presence of a neurodevelopmental impairment. In this framework, in line with the strong genetic heritability associated with ASD, it is also possible that alcohol exposure should not be considered as a potential causative factor but as one of the environmental factors which may interact, during intra-uterine life, with genetic vulnerability in promoting ASD development [20,24]. Noticeably, the role of the intra-uterine environment in modulating genetic vulnerability was recently highlighted in a pivotal study on schizophrenia [124].

This review should be considered in light of several methodological limitations.

Firstly, this is a narrative review. Thus, by definition, it was not led according to a systematic protocol for study selection. As a result, a systematic diagram flow with included/excluded studies, which would have facilitated the replicability of the procedure, is not available, eventually opening the way to the risk of biases. This issue may have led to several biases in the literature selection, including the influence of the authors’ viewpoints, gaps in the literature due to the specific searching practices or eventual errors in the data translation from the primary literature. In order to limit the impact of these limitations, articles have been separately searched and selected by different authors, and eventual discrepancies in the selection have been discussed.

In addition, it should be noted that most of the revised works were five years old or older. Considering the limited number of recent contributions, we chose not to exclude older studies in order to reach a comprehensive overview of the state of the art in the field. However, findings from older studies should be considered cautiously because they could be based on an outdated rationale and not reflect the current understanding in the field.

## 8. Conclusions

The aim of this work was to review the available studies on the possible link between ASD, FASD and in utero alcohol exposure in order to evaluate if the literature would allow reaching some conclusive understanding in the field. However, as described above, the revised studies about epidemiological and symptomatological overlaps between ASD and FASD are scant in number and also report several methodological issues and controversial results. As a consequence, it should be stated that the available data in the scientific literature still does not allow reaching a conclusive remark on the topic. Further research with more rigorous protocols is needed; however, the eventual relationship between FASD and ASD, even if present, may not be easily demonstrable due to the methodological issues reported in the previous sections [18]. Moreover, although symptomatological overlaps between ASD and FASD have been frequently reported, and some similarities in biological underpinnings might be detectable, these findings did not clarify if the similarities could be related to a specific association between the two conditions or simply to the fact that both the disorders feature a neurodevelopmental impairment [21,36,45,77,78,79,88]. In conclusion, the eventual role of alcohol exposure in ASD development and the possible link between ASD and FASD still needs to be clarified. On the other hand, more attention should be paid to identifying these conditions, in particular when clinical presentations are milder or subthreshold. Further research in this field may allow reaching a better understanding of the pathophysiology of neurodevelopmental alterations. From a clinical point of view, further studies may allow identifying more targeted intervention strategies for both ASD and FASD populations.

## Figures and Tables

**Table 1:** Studies focusing on in utero alcohol exposure among subjects already diagnosed with ASD.

Reference	Participants	Main Findings
[26] Williams et al. (2003)	102 AU 106 DD	No association between mother alcohol intake and autism.
[27] Visser et al. (2012)	121 ASD 75 DD 311 controls	No association between mother alcohol intake and autism.
[28] Singer A.B. (2017) SEED Study	684 ASD 869 DD 962 controls from the general population	No association between low levels of maternal alcohol intake and ASD or DD. An inverse association was found in the preconception period and in the third trimester.

Autistic subjects: AU; developmentally disabled: DD; autism spectrum disorder: ASD; Study to Explore Early Development: SEED.

**Table 2:** Studies on the prevalence of ASD diagnosis in children with different levels of alcohol prenatal exposure or FASD.

Reference	Participants	Main Findings
[29] Habbick et al. (1996)	207 FAS	7 cases of ASD
[30] Aronson et al. (1997)	24 children from mothers with alcohol abuse during pregnancy	3 cases of ASD: 2 with Asperger’s Syndrome and another with autistic-like condition 10 cases of ADHD 6 cases of mental retardation
[31] O’Connor et al. (2002)	23 children (with in utero alcohol exposure and with IQ equal to or greater than 70)	No child with ASD 17 cases of ARND 6 cases of FAS/partial FAS
[32] Green et al. (2009)	97 FASD 92 controls	2 cases of ASD in FASD group Children with FASD demonstrated deficits in planning and spatial working memory
[33] Bell et al. (2010)	425 FASD from an original sample of 1063 subjects	8 cases of ASD
[34] Landgren et al. (2010)	71 children adopted from eastern Europe	37 cases of FASD 6 cases of autism 36 cases of ADHD
[35] Eliasen et al. (2010)	80552 Danish children and their mothers	401 cases of ASD 157 cases of infantile autism No association between ASD and average alcohol consumption Binge drinking during pregnancy was associated with a lower risk of ASD
[36] Steven et al. (2013)	25 FASD 17 controls	Higher prevalence of autistic traits in FASD children
[37] Chasnoff et al. (2015)	547 foster or adopted youth referred to a mental health center	156 cases of FASD; among them, 8 cases (5%) with autism/pervasive development disorder High rate of previous FASD missed diagnosis/ misdiagnosis
[38] Mukherjee et al. (2016)	21 FASD	16 cases (72%) of ASD
[40] Gallagher et al. (2018)	18168 mother-child pairs	No significant association between ASD in children and alcohol intake.
[39] Mukherjee et al. (2019)	99 FASD	68% of the sample with ASD or social communication disorder.

Fetal alcohol syndrome: FAS; autism spectrum disorder: ASD; attention deficit hyperactivity disorder: ADHD; intelligence quotient: IQ; alcohol-related neurobehavioral disorders: ARND, fetal alcohol spectrum disorder: FASD.

**Table 3:** Similarities between FASD and ASD children.

Reference	Participants	Main Findings
[41] Olson et al. (1998)	174 adolescents with minimal or no prenatal alcohol exposure 9 non-retarded FAS adolescents 46 controls with IQ range similar to FAS group	FAS adolescents showed more behavioral problems and poorer social abilities with respect to subjects with similar IQ
[46] Thomas et al. (1998)	15 FAS children 15 IQ-matched controls 15 average IQ controls	Social deficits in children with FASD compared to controls matched for verbal IQ.
[47] Mattson & Riley (2000)	55 pre-natal alcohol exposure children (FAS = 35; PAE = 20) 33 non-exposed children	Prenatal alcohol exposure results in significant impairment of parent-rated behaviors, including social problems
[49] Steinhause et al. (2003)	12 children with moderate-to-severe FASD 26 children with mild FASD or FAE 15 control children with unspecific intellectual disability	FASD subjects reported more communication disturbance, anxiety, disruptive, self-absorbed and antisocial behavior
[42] Bishop et al. (2007)	29 AU 33 PDD-NOS 29 FASD	Problems with social interaction and non-verbal communication were detected in the ASD group. Socially inappropriate behaviors and difficulty with peers were highlighted in ASD and FASD groups
[45] Rasmussen et al. (2009).	25 children with FASD 28 controls	Children with FASD have difficulty with theory of mind tasks; theory of mind performance was correlated with executive functions
[36] Stevens et al. (2013).	25 children with FASD 17 controls	FASD reported lower social skills and higher behavioral problems and autistic features
[55] Lidstone et al. (2020)	22 children with ASD, 17 children with FASD, 18 children with ADHD, and 22 TD children	Significantly worse non-dominant hand manual dexterity in ASD than in FASD and TD; significantly worse dominant hand manual dexterity in ASD than in TD; significantly lower hand performance asymmetry in FASD than in ASD and ADHD
[56] Mughal et al. (2020).	29 children with FASD, 21 children with ASD, 46 TD children	Shorter total sleep duration, lower sleep efficiency, and more nocturnal wakings in FASD and ASD than in TD peers. Significant association between sleep and scores reported on the cognitive tests in all groups

Fetal alcohol syndrome: FAS; intelligence quotient: IQ; prenatal alcohol exposure: PAE; fetal alcohol spectrum disorders: FASD; fetal alcohol effects: FAE; autistic subjects: AU; pervasive developmental disorder—not otherwise specified: PDD-NOS; attention deficit hyperactivity disorder: ADHD; typically developing: TD.

## Data Availability

Not applicable.

## References

[1] American Psychiatric Association (2013). Diagnostic and Statistical Manual of Mental Disorders.

[2] Riley E.P., Infante M.A., Warren K.R. (2011). Fetal Alcohol Spectrum Disorders: An Overview. Neuropsychol. Rev..

[3] Burd L., Hofer R. (2008). Biomarkers for detection of prenatal alcohol exposure: A critical review of fatty acid ethyl esters in meconium. Birth Defects Res. A Clin. Mol. Teratol..

[4] Sampson P.D., Streissguth A.P., Bookstein F.L., Little R.E., Clarren S.K., Dehaene P., Hanson J.W., Graham J.M. (1997). Incidence of fetal alcohol syndrome and prevalence of alcohol-related neurodevelopmental disorder. Teratology.

[5] Bagheri M.M., Burd L., Martsolf J.T., Klug M.G. (1998). Fetal alcohol syndrome: Maternal and neonatal characteristics. J. Périnat. Med..

[6] Day N.L., Goldschmidt L., Robles N., Richardson G., Cornelius M., Taylor P., Geva D., Stoffer D. (1991). Prenatal Alcohol Exposure and Offspring Growth at 18 Months of Age: The Predictive Validity of Two Measures of Drinking. Alcohol. Clin. Exp. Res..

[7] May P.A., Gossage J.P., Kalberg W.O., Robinson L.K., Buckley D., Manning M., Hoyme H.E. (2009). Prevalence and epidemiologic characteristics of FASD from various research methods with an emphasis on recent in-school studies. Dev. Disabil. Res. Rev..

[8] Abel E.L., Sokol R.J. (1986). Fetal alcohol syndrome is now leading cause of mental retardation. Lancet.

[9] Popova S., Lange S., Probst C., Gmel G., Rehm J. (2017). Estimation of national, regional, and global prevalence of alcohol use during pregnancy and fetal alcohol syndrome: A systematic review and meta-analysis. Lancet Glob. Health.

[10] Nash A., Davies L. (2017). Fetal Alcohol Spectrum Disorders: What Pediatric Providers Need to Know. J. Pediatr. Health Care.

[11] Hutson J.R., Magri R., Gareri J.N., Koren G. (2010). The Incidence of Prenatal Alcohol Exposure in Montevideo Uruguay as Determined by Meconium Analysis. Ther. Drug Monit..

[12] Gareri J., Lynn H., Handley M., Rao C., Koren G. (2008). Prevalence of Fetal Ethanol Exposure in a Regional Population-Based Sample by Meconium Analysis of Fatty Acid Ethyl Esters. Ther. Drug Monit..

[13] Bakhireva L.N., Sharkis J., Shrestha S., Miranda-Sohrabji T.J., Williams S.R.C.M. (2017). Prevalence of Prenatal Alcohol Exposure in the State of Texas as Assessed by Phosphatidylethanol in Newborn Dried Blood Spot Specimens. Alcohol Clin. Exp. Res..

[14] Streissguth A.P., Dehaene P. (1993). Fetal alcohol syndrome in twins of alcoholic mothers: Concordance of diagnosis and IQ. Am. J. Med. Genet..

[15] Folstein S., Rutter M. (1977). Infantile autism: A genetic study of 21 twin pairs. J. Child Psychol. Psychiatry.

[16] Zhang X., Lv C.-C., Tian J., Miao R.-J., Xi W., Hertz-Picciotto I., Qi L. (2010). Prenatal and Perinatal Risk Factors for Autism in China. J. Autism Dev. Disord..

[17] Carpita B., Carmassi C., Calderoni S., Muti D., Muscarella A., Massimetti G., Cremone I.M., Gesi C., Conti E., Muratori F. (2020). The broad autism phenotype in real-life: Clinical and functional correlates of autism spectrum symptoms and rumination among parents of patients with autism spectrum disorder. CNS Spectr..

[18] Lyall K., Schmidt R.J., Hertz-Picciotto I. (2014). Maternal lifestyle and environmental risk factors for autism spectrum disorders. Int. J. Epidemiol..

[19] Van De Bor M. (2019). Fetal Toxicology. Handb. Clin. Neurol..

[20] Carpita B., Muti D., Dell’Osso L. (2018). Oxidative Stress, Maternal Diabetes, and Autism Spectrum Disorders. Oxid. Med. Cell Longev..

[21] Carpita B., Marazziti D., Palego L., Giannaccini G., Betti L., Dell’Osso L. (2020). Microbiota, Immune System and Autism Spectrum Disorders: An Integrative Model towards Novel Treatment Options. Curr. Med. Chem..

[22] Casanova M.F. (2007). The Neuropathology of Autism. Brain Pathol..

[23] Trottier G., Srivastava L., Walker C.D. (1999). Etiology of infantile autism: A review of recent advances in genetic and neurobiological research. J. Psychiatry Neurosci..

[24] Dell’Osso L., Lorenzi P., Carpita B. (2019). Autistic traits and illness trajectories. Clin Pract. Epidemiol Men Health.

[25] Lange S., Rehm J., Anagnostou E., Popova S. (2018). Prevalence of externalizing disorders and Autism Spectrum Disorders among children with Fetal Alcohol Spectrum Disorder: Systematic review and meta-analysis. Biochem. Cell Biol..

[26] Williams G., Oliver J.M., Allard A.M., Sears L. (2003). Autism and Associated Medical and Familial Factors: A Case Control Study. J. Dev. Phys. Disabil..

[27] Visser J.C., Rommelse N., Vink L., Schrieken M., Oosterling I.J., Van Der Gaag R.J., Buitelaar J.K. (2013). Narrowly Versus Broadly Defined Autism Spectrum Disorders: Differences in Pre- and Perinatal Risk Factors. J. Autism Dev. Disord..

[28] Singer A.B., Aylsworth A.S., Cordero C., Croen L.A., DiGuiseppi C., Fallin M.D., Herring A.H., Hooper S.R., Pretzel R.E., Schieve L.A. (2017). Prenatal Alcohol Exposure in Relation to Autism Spectrum Disorder: Findings from the Study to Explore Early Development (SEED). Paediatr. Périnat. Epidemiol..

[29] Habbick B.F., Nanson J.L., Snyder R.E., Casey R.E., Schulman A.L. (1996). Foetal alcohol syndrome in Saskatchewan: Unchanged incidence in a 20-year period. Can. J. Public Health.

[30] Aronson M., Hagberg B., Gillberg C. (1997). Attention deficits and autistic spectrum problems in children exposed to alcohol during gestation: A follow-up study. Dev. Med. Child Neurol..

[31] O’Connor M.J., Shah B., Whaley S., Cronin P., Gunderson B., Graham J. (2002). Psychiatric Illness in a Clinical Sample of Children with Prenatal Alcohol Exposure. Am. J. Drug Alcohol Abus..

[32] Green C.R., Mihic A.M., Nikkel S.M., Stade B.C., Rasmussen C., Munoz D.P., Reynolds J.N. (2009). Executive function deficits in children with fetal alcohol spectrum disorders (FASD) measured using the Cambridge Neuropsychological Tests Automated Battery (CANTAB). J. Child Psychol. Psychiatry.

[33] Bell S.H., Stade B., Reynolds J.N., Rasmussen C., Andrew G., Hwang P.A., Carlen P.L. (2010). The Remarkably High Prevalence of Epilepsy and Seizure History in Fetal Alcohol Spectrum Disorders. Alcohol. Clin. Exp. Res..

[34] Landgren M., Svensson L., Strömland K., Grönlund M.A. (2010). Prenatal Alcohol Exposure and Neurodevelopmental Disorders in Children Adopted from Eastern Europe. Pediatrics.

[35] Eliasen M.H., Tolstrup J.S., Andersen A.-M.N., Grønbaek M., Olsen J., Strandberg-Larsen K. (2010). Prenatal alcohol exposure and autistic spectrum disorders-a population-based prospective study of 80 552 children and their mothers. Int. J. Epidemiol..

[36] Stevens S.A., Nash K., Koren G., Rovet J. (2013). Autism characteristics in children with fetal alcohol spectrum disorders. Child Neuropsychol..

[37] Chasnoff I.J., Wells A.M., King L. (2015). Misdiagnosis and Missed Diagnoses in Foster and Adopted Children with Prenatal Alcohol Exposure. Pediatrics.

[38] Mukherjee R.A., Layton M., Yacoub E., Turk J. (2011). Autism and autistic traits in people exposed to heavy prenatal alcohol: Data from a clinical series of 21 individuals and nested case control study. Adv. Ment. Health Intellect. Disabil..

[39] Mukherjee R.A., Cook P.A., Norgate S.H., Price A.D. (2019). Neurodevelopmental outcomes in individuals with fetal alcohol spectrum disorder (FASD) with and without exposure to neglect: Clinical cohort data from a national FASD diagnostic clinic. Alcohol.

[40] Gallagher C., McCarthy F.P., Ryan R.M., Khashan A.S. (2018). Maternal Alcohol Consumption During Pregnancy and the Risk of Autism Spectrum Disorders in Offspring: A Retrospective Analysis of the Millennium Cohort Study. J. Autism Dev. Disord..

[41] Olson H.C., Feldman J.J., Streissguth A.P., Sampson P.D., Bookstein F.L. (1998). Neuropsychological deficits in adolescents with fetal alcohol syndrome: Clinical findings. Alcohol. Clin. Exp. Res..

[42] Bishop S., Gahagan S., Lord C. (2007). Re-examining the core features of autism: A comparison of autism spectrum disorder and fetal alcohol spectrum disorder. J. Child Psychol. Psychiatry.

[43] Baron-Cohen S., Leslie A.M., Frith U.T.A. (1985). Does the autistic child have a “theory of mind”?. Cognition.

[44] Hoogenhout M., Malcolm-Smith S. (2017). Theory of mind predicts severity level in autism. Autism.

[45] Rasmussen C., Wyper K., Talwar V. (2009). The relation between theory of mind and executive functions in children with fetal alcohol spectrum disorders. Can. J. Clin. Pharmacol. J..

[46] Thomas S.E., Kelly S.J., Mattson S.N., Riley E.P. (1998). Comparison of social abilities of children with fetal alcohol syndrome to those of children with similar IQ scores and normal controls. Alcohol Clin. Exp. Res..

[47] Mattson S.N., Riley E.P. (2000). Parent ratings of behavior in children with heavy prenatal alcohol exposure and IQ-matched controls. Alcohol Clin. Exp. Res..

[48] Burd L., Klug M.G., Martsolf J.T., Kerbeshian J. (2003). Fetal alcohol syndrome: Neuropsychiatric phenomics. Neurotoxicol. Teratol..

[49] Steinhausen H.-C., Willms J., Metzke C.W., Spohr H.-L. (2003). Behavioural phenotype in foetal alcohol syndrome and foetal alcohol effects. Dev. Med. Child Neurol..

[50] Ikonomidou C., Bittigau P., Ishimaru M.J., Wozniak D.F., Koch C., Genz K., Price M.T., Stefovska V., Hörster F., Tenkova T. (2000). Ethanol-Induced Apoptotic Neurodegeneration and Fetal Alcohol Syndrome. Science.

[51] Middleton F.A., Varlinskaya E., Mooney S.M. (2012). Molecular Substrates of Social Avoidance Seen following Prenatal Ethanol Exposure and Its Reversal by Social Enrichment. Dev. Neurosci..

[52] Bauminger N., Solomon M., Rogers S.J. (2010). Externalizing and internalizing behaviors in ASD. Autism Res..

[53] Mahan S., Matson J.L. (2011). Children and adolescents with autism spectrum disorders compared to typically developing controls on the Behavioral Assessment System for Children, Second Edition (BASC-2). Res. Autism Spectr. Disord..

[54] Leyfer O.T., Folstein S.E., Bacalman S., Davis N.O., Dinh E., Morgan J., Tager-Flusberg H., Lainhart J.E. (2006). Comorbid Psychiatric Disorders in Children with Autism: Interview Development and Rates of Disorders. J. Autism Dev. Disord..

[55] Lidstone D.E., Miah F.Z., Poston B., Beasley J.F., Dufek J.S. (2020). Manual dexterity in children with autism spectrum disorder: A cross-syndrome approach. Res. Autism Spectr. Disord..

[56] Mughal R., Hill C.M., Joyce A., Dimitriou D. (2020). Sleep and Cognition in Children with Fetal Alcohol Spectrum Disorders (FASD) and Children with Autism Spectrum Disorders (ASD). Brain Sci..

[57] Atta C.A.M., Fiest K.M., Frolkis A.D., Jette N., Pringsheim T., St Germaine-Smith C., Rajapakse T., Kaplan G.G., Metcalfe A. (2016). Global birth prevalence of spina bifida by folic acid fortification status: A systematic review and meta-analysis. Am. J. Public Health.

[58] Hamid A., Wani N.A., Kaur J. (2009). New perspectives on folate transport in relation to alcoholism-induced folate malabsorption—association with epigenome stability and cancer development. FEBS J..

[59] Gupta K.K., Gupta V.K., Shirasaka T. (2016). An Update on Fetal Alcohol Syndrome—Pathogenesis, Risks, and Treatment. Alcohol Clin. Exp. Res..

[60] Kapur B.M., Baber M. (2018). FASD: Folic acid and formic acid—An unholy alliance in the alcohol abusing mother. Biochem. Cell Biol..

[61] Hutson J.R., Stade B., Lehotay D.C., Collier C.P., Kapur B.M. (2012). Folic Acid Transport to the Human Fetus Is Decreased in Pregnancies with Chronic Alcohol Exposure. PLoS ONE.

[62] Schmidt R., Hansen R.L., Hartiala J., Allayee H., Schmidt L.C., Tancredi D., Tassone F., Hertz-Picciotto I. (2011). Prenatal Vitamins, One-carbon Metabolism Gene Variants, and Risk for Autism. Epidemiology.

[63] Surén P., Roth C., Bresnahan M., Haugen M., Hornig M., Hirtz D., Lie K.K., Lipkin W.I., Magnus P., Reichborn-Kjennerud T. (2013). Association Between Maternal Use of Folic Acid Supplements and Risk of Autism Spectrum Disorders in Children. JAMA.

[64] Hoxha B., Hoxha M., Domi E., Gervasoni J., Persichilli S., Malaj V., Zappacosta B. (2021). Folic Acid and Autism: A Systematic Review of the Current State of Knowledge. Cells.

[65] Virk J., Liew Z., Olsen J., Nohr E., Catov J.M., Ritz B. (2015). Preconceptional and prenatal supplementary folic acid and multivitamin intake and autism spectrum disorders. Autism.

[66] Egorova O., Myte R., Schneede J., Hägglöf B., Bölte S., Domellöf E., Ivars A’Roch B., Elgh F., Ueland P.M., Silfverdal S.A. (2020). Maternal blood folate status during early pregnancy and occurrence of autism spectrum disorder in offspring: A study of 62 serum biomarkers. Mol. Autism.

[67] Ramaekers V.T., Rothenberg S.P., Sequeira J.M., Opladen T., Blau N., Quadros E.V., Selhub J. (2005). Autoantibodies to Folate Receptors in the Cerebral Folate Deficiency Syndrome. N. Engl. J. Med..

[68] Hertz-Picciotto I., Schmidt R., Krakowiak P. (2018). Understanding environmental contributions to autism: Causal concepts and the state of science. Autism Res..

[69] Varadinova M., Boyadjieva N. (2015). Epigenetic mechanisms: A possible link between autism spectrum disorders and fetal alcohol spectrum disorders. Pharmacol. Res..

[70] Schanen N.C. (2006). Epigenetics of autism spectrum disorders. Hum. Mol. Genet..

[71] Liu Y., Balaraman Y., Wang G., Nephew K.P., Zhou F.C. (2009). Alcohol exposure alters DNA methylation profiles in mouse embryos at early neurulation. Epigenetics.

[72] Garro A.J., McBeth D.L., Lima V., Lieber C.S. (1991). Ethanol Consumption Inhibits Fetal DNA Methylation in Mice: Implications for the Fetal Alcohol Syndrome. Alcohol Clin. Exp. Res..

[73] Haycock P.C. (2009). Fetal Alcohol Spectrum Disorders: The Epigenetic Perspective1. Biol. Reprod..

[74] Laufer B.I., Mantha K., Kleiber M.L., Diehl E.J., Addison S.M.F., Singh S.M. (2013). Long-lasting alterations to DNA methylation and ncRNAs could underlie the effects of fetal alcohol exposure in mice. Dis. Model. Mech..

[75] Hicks S.D., Middleton F.A., Miller M.W. (2010). Ethanol-induced methylation of cell cycle genes in neural stem cells. J. Neurochem..

[76] Perkins A., Lehmann C., Lawrence R.C., Kelly S.J. (2013). Alcohol exposure during development: Impact on the epigenome. Int. J. Dev. Neurosci..

[77] Banik A., Kandilya D., Ramya S., Stünkel W., Chong Y.S., Dheen S.T. (2017). Maternal Factors that Induce Epigenetic Changes Contribute to Neurological Disorders in Offspring. Genes.

[78] Samaco R.C., Nagarajan R.P., Braunschweig D., LaSalle J.M. (2004). Multiple pathways regulate MeCP2 expression in normal brain development and exhibit defects in autism-spectrum disorders. Hum. Mol. Genet..

[79] Nagarajan R.P., Hogart A.R., Gwye Y., Martin M.R., LaSalle J.M. (2006). Reduced MeCP2 Expression is Frequent in Autism Frontal Cortex and Correlates with Aberrant MECP2 Promoter Methylation. Epigenetics.

[80] Zhou F.C., Balaraman Y., Teng M., Liu Y., Singh R.P., Nephew K.P. (2011). Alcohol Alters DNA Methylation Patterns and Inhibits Neural Stem Cell Differentiation. Alcohol. Clin. Exp. Res..

[81] Zhou F.C., Zhao Q., Liu Y., Goodlett C.R., Liang T., McClintick J.N., Edenberg H.J., Li L. (2011). Alteration of gene expression by alcohol exposure at early neurulation. BMC Genom..

[82] Hof P.R., Knabe R., Bovier P., Bouras C. (1991). Neuropathological observations in a case of autism presenting with self-injury behavior. Acta Neuropathol..

[83] Masi A., Quintana D.S., Glozier N., Lloyd A.R., Hickie I.B., Guastella A.J. (2015). Cytokine aberrations in autism spectrum disorder: A systematic review and meta-analysis. Mol. Psychiatry.

[84] Vargas D.L., Nascimbene C., Krishnan C., Zimmerman A.W., Pardo C.A. (2005). Neuroglial activation and neuroinflammation in the brain of patients with autism. Ann. Neurol..

[85] Savino R., Carotenuto M., Polito A., Di Noia S., Albenzio M., Scarinci A., Ambrosi A., Sessa F., Tartaglia N., Messina G. (2020). Analyzing the Potential Biological Determinants of Autism Spectrum Disorder: From Neuroinflammation to the Kynurenine Pathway. Brain Sci..

[86] Dufour-Rainfray D., Vourc’H P., Tourlet S., Guilloteau D., Chalon S., Andres C.R. (2011). Fetal exposure to teratogens: Evidence of genes involved in autism. Neurosci. Biobehav. Rev..

[87] Bhatia S., Drake D.M., Miller L., Wells P.G. (2019). Oxidative stress and DNA damage in the mechanism of fetal alcohol spectrum disorders. Birth Defects Res..

[88] Brocardo P.S., Gil-Mohapela J., Christiea B.R. (2011). The role of oxidative stress in fetal alcohol spectrum disorders. Brain. Res. Rev..

[89] Goodlett C.R., Horn K.H., Zhou F.C. (2011). Alcohol Teratogenesis: Mechanisms of Damage and Strategies for Intervention. Exp. Biol. Med..

[90] Luo J. (2014). Autophagy and ethanol neurotoxicity. Autophagy.

[91] Ehrhart F., Roozen S., Verbeek J., Koek G., Kok G., Van Kranen H., Evelo C., Curfs L.M.G. (2017). Review and gap analysis: Molecular pathways leading to fetal alcohol spectrum disorders. Mol. Psychiatry.

[92] Da Lee R., An S.M., Kim S.S., Rhee G.S., Kwack S.J., Seok J.H., Chae S.Y., Park C.H., Choi Y.W., Kim H.S. (2005). Neurotoxic Effects of Alcohol and Acetaldehyde During Embryonic Development. J. Toxicol. Environ. Health A.

[93] Ahluwalia B., Wesley B., Adeyiga O., Smith D.M., Da-Silva A., Rajguru S. (2000). Alcohol modulates cytokine secretion and synthesis in human fetus: An in vivo and in vitro study. Alcohol.

[94] Drew D.P., Johnson J.W., Douglas C.J., Phelen D.K., Kane J.M.C. (2015). Pioglitazone Blocks Ethanol Induction of Microglial Activation and Immune Responses in the Hippocampus, Cerebellum, and Cerebral Cortex in a Mouse Model of Fetal Alcohol Spectrum Disorders. Alcohol Clin. Exp. Res..

[95] Sowell K., Uriu-Adams J., Van de Water J., Chambers C.D., Coles C., Kable J., Yevtushok L., Zymak-Zakutnya N., Wertelecki W., Keen C. (2017). Implications of altered maternal cytokine concentrations on infant outcomes in children with prenatal alcohol exposure. Alcohol.

[96] King C.R. (2011). A novel embryological theory of autism causation involving endogenous biochemicals capable of initiating cellular gene transcription: A possible link between twelve autism risk factors and the autism ‘epidemic’. Med. Hypotheses.

[97] Iwasa F., Galbraith R.A., Sassa S. (1988). Effects of dimethyl sulphoxide on the synthesis of plasma proteins in the human hepatoma HepG2. Induction of an acute-phase-like reaction. Biochem. J..

[98] Reynolds J.D., Brien F.J. (1994). Ethanol neurobehavioural teratogenesisnand the role of bglutamate in the fetal hippocampus. Candian J. Physiol. Pharm..

[99] McDonald J.W., Johnston M.V. (1990). Physiological and pathophysiological roles of excitatory amino acids during central nervous system development. Brain Res. Rev..

[100] Samuelson D.R., Gu M., Shellito J.E., Molina P.E., Taylor C.M., Luo M., Welsh D.A. (2019). Intestinal Microbial Products from Alcohol-Fed Mice Contribute to Intestinal Permeability and Peripheral Immune Activation. Alcohol. Clin. Exp. Res..

[101] Bajaj J.S. (2019). Alcohol, liver disease and the gut microbiota. Nat. Rev. Gastroenterol. Hepatol..

[102] Duan Y., Llorente C., Lang S., Brandl K., Chu H., Jiang L., White R.C., Clarke T.H., Nguyen K., Torralba M. (2019). Bacteriophage targeting of gut bacterium attenuates alcoholic liver disease. Nature.

[103] Szabo G. (2015). Gut–Liver Axis in Alcoholic Liver Disease. Gastroenterology.

[104] Pascual M., Montesinos J., Montagud-Romero S., Forteza J., Rodríguez-Arias M., Miñarro J., Guerri C. (2017). TLR4 response mediates ethanol-induced neurodevelopment alterations in a model of fetal alcohol spectrum disorders. J. Neuroinflamm..

[105] Shukla P.K., Meena A.S., Rao R., Rao R.K. (2018). Deletion of TLR-4 attenuates fetal alcohol exposure-induced gene expression and social interaction deficits. Alcohol.

[106] Virdee M.S., Saini N., Kay C.D., Neilson A.P., Kwan S.T.C., Helfrich K.K., Mooney S.M., Smith S.M. (2021). An enriched biosignature of gut microbiota-dependent metabolites characterizes maternal plasma in a mouse model of fetal alcohol spectrum disorder. Sci. Rep..

[107] Hsiao E.Y., McBride S.W., Hsien S., Sharon G., Hyde E.R., McCue T., Codelli J.A., Chow J., Reisman S.E., Petrosino J.F. (2013). Microbiota Modulate Behavioral and Physiological Abnormalities Associated with Neurodevelopmental Disorders. Cell.

[108] Cao X., Lin P., Jiang P., Li C. (2013). Characteristics of the gastrointestinal microbiome in children with autism spectrum disorder: A systematic review. Shanghai Arch. Psychiatry.

[109] Coles C.D. (2011). Discriminating the effects of prenatal alcohol exposure from other behavioral and learning disorders. Alcohol Res. Health.

[110] Seagull F.N., Mowery J.L., Simpson P.M., Robinson T.R., Martier S.S., Sokol R.J., McCarver-May D.G. (1996). Maternal assessment of infant development: Associations with alcohol and drug use in pregnancy. Clin. Pediat..

[111] Benz J., Rasmussen C., Andrew G. (2009). Diagnosing fetal alcohol spectrum disorder: History, challenges and future directions. Paediatr. Child Health.

[112] Kesmodel U., Olsen S.F. (2001). Self reported alcohol intake in pregnancy: Comparison between four methods. J. Epidemiol. Community Health.

[113] Streissguth A.P., Bookstein F.L., Barr H.M., Sampson P.D., O’Malley K., Young J.K. (2004). Risk factors for adverse life outcomes in Fetal Alcohol Sydnrome and Fetal Alcohol Effects. J. Dev. Behav. Ped..

[114] McLennan J.D. (2015). Misattributions and Potential Consequences: The Case of Child Mental Health Problems and Fetal Alcohol Spectrum Disorders. Can. J. Psychiatry.

[115] Stockwell T., Donath S., Cooper-Stanbury M., Chikritzhs T., Catalano P., Mateo C. (2004). Under-reporting of alcohol consumption in household surveys: A comparison of quantity-frequency, graduated-frequency and recent recall. Addiction.

[116] Tau G.Z., Peterson B.S. (2010). Normal development of brain circuits. Neuropsychopharmacology.

[117] De Rubeis S., He X., Goldberg A.P., Poultney C.S., Samocha K., Cicek A.E., Kou Y., Liu L., Fromer M., Walker S. (2014). Synaptic, transcriptional and chromatin genes disrupted in autism. Nature.

[118] Arndt T.L., Stodgell C.J., Rodier P.M. (2005). The teratology of autism. Int. J. Dev. Neurosci..

[119] Lobascher M.E., Kingerlee P.E., Gubbay S.S. (1970). Childhood autism: An investigation of aetiological factors in twenty-five cases. Br. J. Psychiatry.

[120] Miles J.H., Takahashi T.N., Haber A., Hadden L. (2003). Autism families with a high incidence of alcoholism. J. Autism Dev. Disord..

[121] Bolton P.F., Pickles A., Murphy M., Rutter M. (1998). Autism, affective and other psychiatric disorders: Patterns of familial aggregation. Psychol. Med..

[122] Robinson P.D., Schutz C.K., Macciardi F., White B.N., Holden J.J.A. (2001). Genetically determined low maternal serum dopamine β-hydroxylase levels and the etiology of autism spectrum disorders. Am. J. Med. Genet..

[123] Lovinger D.M., Sung K.W., Zhou Q. (2000). Ethanol and trichloroethanol alter gating of 5-HT3 receptor-channels in NCB-20 neuroblastoma cells. Neuropharmacology.

[124] Ursini G., Punzi G., Chen Q., Marenco S., Robinson J.F., Porcelli A., Hamilton E.G., Mitjans M., Maddalena G., Begemann M. (2018). Convergence of placenta biology and genetic risk for schizophrenia. Nat. Med..

